# Zfp422 promotes skeletal muscle differentiation by regulating EphA7 to induce appropriate myoblast apoptosis

**DOI:** 10.1038/s41418-019-0448-9

**Published:** 2019-11-04

**Authors:** Yaping Nie, Shufang Cai, Renqiang Yuan, Suying Ding, Xumeng Zhang, Luxi Chen, Yaosheng Chen, Delin Mo

**Affiliations:** State Key Laboratory of Biocontrol, School of Life Sciences, Sun Yat-sen University, Guangzhou, Guangdong China

**Keywords:** Cell biology, Molecular biology

## Abstract

Zinc finger protein 422 (Zfp422) is a widely expressed zinc finger protein that serves as a transcriptional factor to regulate downstream gene expression, but until now, little is known about its roles in myogenesis. We found here that Zfp422 plays a critical role in skeletal muscle development and regeneration. It highly expresses in mouse skeletal muscle during embryonic development. Specific knockout of Zfp422 in skeletal muscle impaired embryonic muscle formation. Satellite cell-specific Zfp422 deletion severely inhibited muscle regeneration. Myoblast differentiation and myotube formation were suppressed in Zfp422-deleted C2C12 cells, isolated primary myoblasts, and satellite cells. Chromatin Immunoprecipitation Sequencing (ChIP-Seq) revealed that Zfp422 regulated ephrin type-A receptor 7 (EphA7) expression by binding an upstream 169-bp DNA sequence, which was proved to be an enhancer of *EphA7*. Knocking EphA7 down in C2C12 cells or deleting Zfp422 in myoblasts will inhibit cell apoptosis which is required for myoblast differentiation. These results indicate that Zfp422 is essential for skeletal muscle differentiation and fusion, through regulating EphA7 expression to maintain proper apoptosis.

## Introduction

Skeletal muscle is the most abundant and highly complex tissue in a normal vertebrate body. The process of vertebrate skeletal muscle development includes the proliferation, cell cycle withdrawal, differentiation, fusion, and elongation into multinucleated myofibers of myogenic precursor cells or myoblasts [1–3].

Adult skeletal muscle has remarkable regenerative capacity, due to the satellite cells locating at the myofibers periphery [4–6]. There are many similarities between developmental and regenerative myogenesis, including transcription factors and signaling molecules in common [7–9], thus regenerative myogenesis could be a decent model to study muscle development.

The transcriptional mechanisms governing skeletal muscle development have been explained by a large amount of studies. MRFs (Myf5, MyoD, Myogenin, and MRF4) are vital muscle specific transcription factors, they bind E-boxes in the promoters as well as enhancers of their target genes and recruit coregulators, RNA polymerase II and chromatin remodeling factors, and they often act in cooperation with other transcription factors, such as MEF2 and E proteins [10–13].

In addition, there are many nontranscription factors playing important roles in regulating cell–cell contact between myoblasts and its surrounding cells [1, 14]. Cell–cell contact, such as cell adhesion, migration [15, 16], and fusion [17], is also vital in skeletal muscle development. A lot of these nontranscription factors are membrane-associated proteins, such as G-protein coupled receptor BAI3 [18], Laminin [19], β1 integrins [20], and newly discovered Myomaker [21]. It is worth mentioning that a phosphatidylserine receptor BAI1 has been reported to promote fusion of healthy myoblasts by receiving signals from apoptotic cells, and apoptosis of myoblasts is necessary cue for fusion [22, 23].

Zinc finger proteins are abundant in eukaryotic cells and are versatile players in biological processes such as cell proliferation, differentiation, regeneration, metabolism, and autophagy [24, 25]. Several Krüppel-like zinc finger proteins are involved in the regulation of skeletal muscle development and function [26, 27]. For instance, Klf5 is reported to be highly expressed in differentiating myoblasts during muscle regeneration, and regulates myogenesis-related genes in collaboration with MyoD and MEF2 [28]. Zfp637 is expressed in proliferating C2C12 myoblasts and is downregulated during myogenic differentiation, it promotes myoblast proliferation but inhibits myogenic differentiation [29].

Zfp422 is a zinc finger protein containing 5 Krüppel-like zinc finger repeats, and also contains several consensus amino acid sequences for a protein kinase C binding domain [30]. During fetal development, Zfp422 is expressed in the epithelial component of the developing dental organs, osteoblasts of developing craniofacial bones as well as in the developing tongue [31, 32], indicating a potential function of Zfp422 in regulating the development of teeth, craniofacial skeleton, and skeletal muscle.

Eph receptors are the largest family of receptor tyrosine kinases, they and their ephrin ligands are anchored to the plasma membrane and form Eph/ephrin signaling with roles in development and disease pathogenesis [33, 34]. They are guidance molecules that regulate cell–cell communications such as adhesion, repulsion, and migration [35–37]. Eph/ephrin signaling has been reported to implicate in cell adhesion, migration, and motility during skeletal muscle development. Multiple Eph receptors and ephrin ligands are strongly upregulated in activated muscle satellite cells and regenerating myofibers, their guidance signaling modulates satellite cell motility and patterning of regenerating muscle [38, 39]. EphA8 and ephrinA3 promote slow myofiber specification, maintenance, repair, and reinnervation [40]. EphrinA5 expression induced by NF-кB in NG2^+^ interstitial cells promotes cell–cell contact and myoblast migration to the ends of growing fibers [16].

EphA7 also plays important roles in various cells. For instance, it enhances human hematopoietic stem and progenitor cell (HSPC) maintenance, migration, and adhesion [41], and induces prostate tumor cell apoptosis to inhibit prostate tumor growth and progression [42]. A recent study about gene expression profiling of muscle stem cells identifies EphA7 as a potential regulator of postnatal myogenesis [43].

In this work, we identified the role Zfp422 played in embryonic skeletal muscle development, adult muscle regeneration, and in vitro myoblast differentiation. This study demonstrates that Zfp422 binds to a DNA site upstream of *EphA7* gene, where lies its enhancer, promotes its expression to maintain proper apoptosis for a fraction of myoblasts during the early stage of differentiation, which is crucial for myoblast differentiation and fusion, and eventually contribute to normal muscle formation. This work not only reveals the physiological function of Zfp422 in vivo, but also further supports the idea that the appropriate amount of apoptosis is beneficial and necessary for living organisms.

## Materials and methods

### Mice

*Zfp422*^*fl/+*^ mice were created via CRISPR/Cas9 system. Firstly, two sgRNAs-targeting the introns on both sides of the floxed region (contains extron2) of *Zfp422* were synthesized and transcribed, respectively. The donor vector with the loxP fragment was designed and constructed in vitro. Then Cas9 mRNA, sgRNA and donor were co-injected into zygotes. Thereafter, the zygotes were transferred into the oviduct of pseudo pregnant ICR females at 0.5 days post coitum, and F0 mice was born 19–21 days after transplantation. All the offspring of ICR females (F0 mice) were identified by PCR and sequencing using tail DNA (Fig. S7). Finally, F0 mice were crossed with C57BL/6J mice to create heterozygous mice, which were used to produce homozygous *Zfp422*^*fl/fl*^ mice.

*Pax7*^*CreET2R/+*^ (stock #017763) and *Myf5*^*Cre/+*^ (stock #007893) mice were purchased from the Jackson Lab. *Zfp422*^*fl/fl*^ mice were crossed with *Pax7*^*CreERT2/+*^ and *Myf5*^*Cre/+*^ mice to generate *Zfp422*^*fl/fl*^*;Pax7*^*CreERT2/+*^ and *Zfp422*^*fl/fl*^*;Myf5*^*Cre/+*^ mice, respectively. All mice used in this study had a C57BL/6J genetic background, and housed in SPF condition during the experiment. All experimental procedures involving mice in this study were approved by the Animal Care and Use Committee of Guangdong Province and carried out in accordance with ethical standards.

### TMX injection and muscle CTX injury

Tamoxifen (Sigma, Shanghai, China) was dissolved in corn oil (Meilun Biotechnology) to a concentration of 20 mg/ml, CTX (Sigma, Shanghai, China) was dissolved in sterile saline to a final concentration of 10 mM. 8–12-week-old *Zfp422*^*fl/fl*^*;Pax7*^*CreERT2/+*^ and *Zfp422*^*fl/fl*^*;Pax7*^*+/+*^ mice were intraperitoneally injected with 5 μl/g of tamoxifen solution daily for 5 days prior to induction of muscle injury. Three days later, to induce muscle regeneration, mice were anesthetized and legs were cleaned with alcohol, tibialis anterior (TA) muscles of mice were intramuscularly injected with 50 μl of CTX by a hypodermic syringe. Regenerating TA muscles were isolated 5, 10, and 180 days after CTX injection.

### Satellite cells and primary myoblasts isolation and culture conditions

Myofiber and satellite cells were isolated based on the method previously described [44]. Briefly, extensor digitorum longus (EDL) of 8-week-old male mice were isolated and digested in 0.2% (wt/vol) collagenase NB 4G (SERVA Electrophoresis, Germany) in Dulbecco’s modified Eagles medium (DMEM, Sigma) in a shaker water bath at 37 °C for 1.5–2 h. Then single-muscle fibers are liberated by repeatedly triturating the muscle with a wide-mouth Pasteur pipette under a stereomicroscope, washed three times in DMEM and then plated on Matrigel (Corning) coated 24-well plate. After attachment, DMEM with 20% fetal bovine serum, 1% penicillin/streptomycin, 1000 U/ml mouse leukocyte inhibitory factor (LIF; eBioscience) and 10 ng/ml human basic fibroblast growth factor (bFGF, CST) was added to each well, then incubated at 37 °C under 5% CO_2_ in a humidified chamber. During the first 4 days in culture, satellite cells detached, migrated from the fiber, then the fiber was removed. On day 8, the culture medium was changed to DMEM with 2% horse serum to induce differentiation.

Primary myoblasts were isolated based on the method previously described [21]. Dorsal muscle were dissected from E17 to E17.5 embryos and dissociated in 1 mg/ml Collagenase type I (Sigma) in DMEM at 37 °C for 1.5–2 h. Ten milliliters of culture media (20% FBS/DMEM) was added to the suspension and triturated followed by centrifugation at 1600 × *g* for 10 min. The pellet was resuspended in 10 ml of growth media (20% FBS/DMEM + 2.5 ng/ml bFGF), filtered through a 100 µm cell strainer, and plated on a 10 cm Matrigel coated culture dish. To enrich for myoblasts, cultures were incubated in a small volume of PBS, and the myoblasts were dislodged by knocking the plate lightly. When cells reached confluence, the culture medium was replaced to DMEM with 2% horse serum to induce differentiation.

C2C12 cells were purchased from ATCC, cultured in DMEM with 10% fetal bovine serum, and 1% penicillin/streptomycin (growth medium, GM) at subconfluent densities. To induce differentiation, cells were switched into DMEM with 2% horse serum 1% penicillin/streptomycin (differentiation medium, DM) after reaching confluence. All cells were maintained in a humidified incubator with 5% CO_2_ at 37 °C.

### Generation of Zfp422 knockout C2C12 cells

*Zfp422* gene was engineered using CRISPR-Cas9 system. In brief, a pair of guide RNAs flanking the mouse *Zfp422* gene was inserted to pUC57-T7-gRNA plasmid vector, then transiently co-transfected into C2C12 cells with Cas9-D10A Nickase plasmid. Deletion of targeted loci was confirmed by DNA sequencing and PCR. Cells transfected with the same plasmid vector but had not deleted *Zfp422* gene were used as a control.

### Plasmids, siRNAs, and transfection

For Zfp422 expression vector, mouse *Zfp4*22 (NCBI Sequence ID: NM_001302439.1) CDS sequence was inserted into pcDNA3.1-Myc-C vector, which modified from pcDNA3.1 vector (Invitrogen, Shanghai, China). For EphA7 expression vector, mouse *EphA7* (NCBI sequence ID: NM_010141.4) CDS sequence was inserted into pcDNA3.1 vector. EphA7 siRNAs and negative control siRNAs were purchased from Invitrogen. C2C12 cells were transfected with plasmids or siRNAs using Lipofectamine 3000 (Invitrogen) according to the manufacturer’s instruction. All transfections were performed in triplicate for each experiment.

### RNA isolation and quantitative real-time PCR

Total RNA was extracted from cultured C2C12 cells, regenerating TA muscles and fetal dorsal muscles using TRI_ZOL_ (Invitrogen) and cDNA was synthesized from 1 μg total RNA by the Reverse Transcription Kit (Promega, Shanghai, China). Quantitative real-time PCR assays were performed on the LightCycler 480 system (Roche, Basel, Switzerland) using SYBR Green qPCR Mix (Dongsheng Biotech, Guangzhou, China), with GAPDH as an internal control for normalization.

### RNA-seq and analysis

Total RNA extracted from Zfp422-KO and control C2C12 cells was enriched by oligo-dT into the polyA+ fraction for sequencing. Sequencing and analysis were performed with an Illumina Genome Analyzer (Illumina, CA) according to the manufacturer’s instructions.

### Luciferase assay

Mouse *EphA7* promoter sequence (Chr4+ :28811762-28813288; Length = 1527 bp) was inserted into *Kpn* I and *Hind* III sites, and +strand or −strand of Zfp422 binding sequence (Chr4+ :28176897-28177065; Length = 169 bp) upstream *EphA7* was inserted to *Bam*H I and *Sal* I, in pGL3-basic vector (Promega). Zfp422 expression vector or pcDNA3.1 together with reconstructed pGL3-basic vector and pRL-TK (Promega) were co-transfected into C2C12 cells or Zfp422-KO C2C12 cells. After 48 h, cells were lysed for analysis of luciferase activities by using the Dual-Luciferase reporter assay system (E1910, Promega) according to the manufacturer’s instruction. The luciferase activity of firefly was normalized to that of Renilla to exclude the differences of transfection efficiency.

### Chromatin immunoprecipitation (ChIP)

ChIP assays were carried out using the Millipore ChIP Assay Kit (Millipore) as per the manufacture’s protocol. In brief, C2C12 cells transfected with 3.1-Zfp422-Myc or 3.1-GFP-Myc were fixed with formaldehyde for 10 min to generate cross-link of protein-DNA complexes. Cell lysates were then sonicated to generate chromatin fragments of 200–300 bp and immunoprecipitated using anti-Myc antibody or IgG as a negative control at 4 °C overnight. Precipitated chromatin DNA was recovered and analyzed by ChIP-seq and PCR.

### ChIP-Seq and analysis

ChIP samples were quantified using a Qubit 2.0 Fluorometer (Invitrogen, Carlsbad, CA, USA) and qualified by Agilent Bioanalyzer 2100 (Agilent Technologies, Palo Alto, CA, USA). Sequencing library preparations were constructed following the manufacturer’s protocol (NEBNext^®^ Ultra™II DNA Library Prep Kit for Illumina^®^). For each sample, at least 10 ng ChIP product was used for library preparation. It was treated with End Prep Enzyme Mix for end repairing, 5′ Phosphorylation and dA-tailing in one reaction, followed by ligation to adapters with a “T” base overhang. Adapter-ligated DNA was then recovered using AxyPrep Mag PCR Clean-up (Axygen). Each sample was then amplified by PCR for eight cycles using P5 and P7 primers, with both primers carrying sequences which can anneal with Flowcell to perform bridge PCR and P7 primer carrying a six-base index allowing for multiplexing. The PCR products were cleaned up using AxyPrep Mag PCR Clean-up, validated using an Agilent 2100 Bioanalyzer, and quantified by Qubit 2.0 Fluorometer. Then libraries with different indexes were multiplexed and loaded on an Illumina HiSeq instrument according to manufacturer’s instructions (Illumina, San Diego, CA, USA). Sequencing was carried out using a 2 × 150 paired-end configuration; image analysis and base calling were conducted by the HiSeq Control Software (HCS) + OLB + GAPipeline-1.6 (Illumina) on the HiSeq instrument.

In order to remove technical sequences, including adapters, PCR primers, or fragments thereof, and quality of bases lower than 20, pass filter data of fastq format were processed by Cutadapt (version 1.9.1) to be high-quality clean data. Clean data were aligned to reference genome via software Bowtie2. Use Homer (V4.6) to analyze peaks quality control, peaks calling, and peaks annotation.

### Western blotting

Protein extracts were obtained by collecting cultured C2C12 cells or mouse TA muscles in lysis buffer (50 mM Tris, 150 mM NaCl, 1% Triton X-100, 0.1% SDS, 1% Sodium deoxycholate, pH 8.0, and freshly added protease inhibitor PMSF), then electrophoresed on SDS-PAGE and transferred to 0.45 μm PVDF membrane (Roche). Membranes were blocked with 3% BSA/TBST for 1–2 h and then incubated with primary antibodies at 4 °C overnight, followed by incubation with proper secondary antibodies. Blots were visualized by a commercial enhanced chemiluminescence (ECL) detection kit (FDbio, China), with GAPDH as an internal control for normalization.

### H&E staining of paraffin sections

Freshly isolated adult TA muscles and fetal dorsal muscles were immediately fixed in 4% paraformaldehyde at 4 °C for 18 h, then performed dehydration by graded ethanol and paraffin embedding, then sectioned by 8 μm, using STP120, EC350, and HM340 (MICROM, Germany) according to the manufacturer’s instruction. TA muscle paraffin sections were rehydrated by graded ethanol and H_2_O and stained using the H&E staining kit (Jiancheng Biotechnology, Nanjing, China) according to the manufacturer’s instruction.

### Immunofluorescence assay

Cultured C2C12 myoblasts, isolated satellite cells, and differentiated myotubes were fixed with 4% paraformaldehyde, permeabilized with 0.5% Triton X-100 in PBS, blocked with 3% BSA/PBS, incubated with primary antibodies at 4 °C overnight, then incubated with proper secondary antibodies for 2 h.

Mouse TA muscle paraffin sections and fetal dorsal muscle paraffin sections were rehydrated by graded ethanol and H_2_O, performed antigen retrieval using citrate antigen retrieval solution, and immunofluorescence was performed using Mouse on the Mouse Polymer IHC Kit (Abcam) according to the manufacturer’s instruction. Observation and images were captured by fluorescent reverse microscopy (Nikon, Japan).

### Flow cytometry analysis for apoptosis

Cells (Zfp422-KO C2C12 cells, control C2C12 cells, and C2C12 cells transfected with siRNA) were induced to differentiate for 1 day, then both floating and adherent C2C12 were harvested and washed by PBS for three times, resuspended and stained with propidium iodide (PI) and annexin V (AnV) using the Annexin V-FITC Apoptosis Detection Kit (Sigma) according to the manufacturer’s instruction. After a 10-min incubation at room temperature, the cells are analyzed by BD FACSCalibur system (BD Biosciences, Franklin Lakes, USA).

To measure caspase-3 activation, C2C12 myoblasts were fixed and permeabilized and stained with Alexa Fluor^®^ 488-conjugated cleaved Caspase-3 (CST) using the Fixation Permeabilization Solution Kit (BD Biosciences), according to the manufacturer’s instruction. Cells were washed in Cytoperm solution, resuspended in 0.5% BSA/PBS and assessed by analyzed by BD FACSCalibur system.

### Quantification and statistics

The differentiation index was calculated as the percentage of nuclei in MyHC-positive cells. The fusion index was calculated as the percentage of nuclei in fused myotubes out of the total nuclei. Over 300 myofibers were evaluated for each mouse (*n* = 3 mice per genotype). Images of H&E staining and immunofluorescence were randomly selected for analysis. Data are presented as mean ± SD or mean from three independent experiments as shown in each graph. Differences between groups were tested for statistical significance using an unpaired two-tailed Student’s *t* test. *P* < 0.05 was considered as significance, except in analysis involving RNA-seq.

## Results

### Zfp422 is essential for embryonic skeletal muscle development

**Fig. 1:**
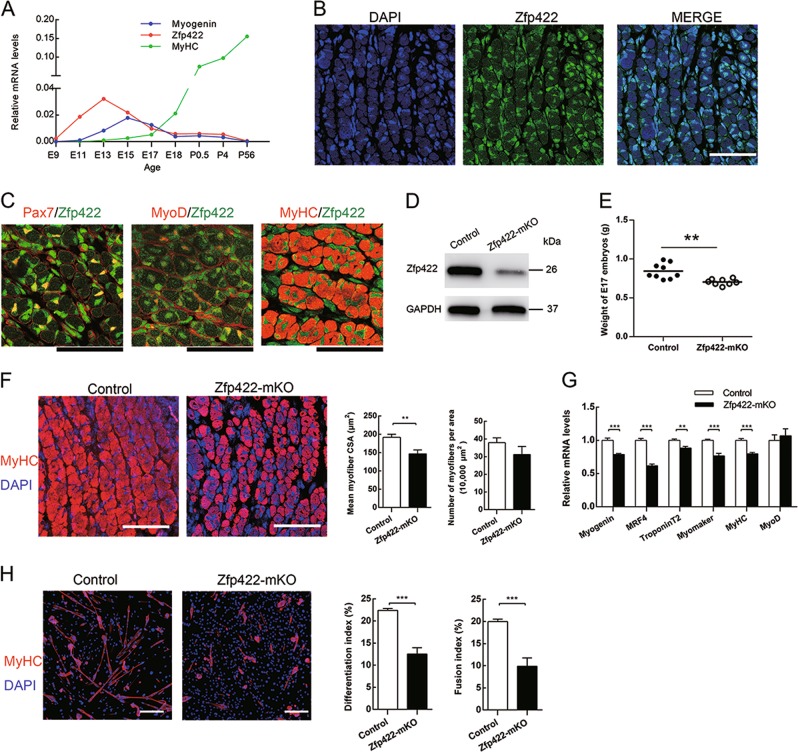
Zfp422 is essential for developmental myogenesis and primary myoblast differentiation. **a** Quantitative PCR for the expression of Zfp422, Myogenin and MyHC in the whole embryo (E9) and dorsal muscle (E11-P56) at the indicated ages. Data are presented as mean *n* = 3 per group (**b**). Staining for Zfp422 and nuclei (DAPI) on the dorsal muscle cross section of WT E17. **c** Co-immunostaining for Zfp422 with markers of progenitors (Pax7), myoblasts (MyoD) and myofibers (MyHC) on dorsal muscle cross sections of WT E17. **d** Zfp422 protein levels in the E17 dorsal muscle of Zfp422+/+;Myf5Cre/+(control) and Zfp422fl/fl;Myf5Cre/+(Zfp422-mKO) embryos. **e** Weight of control and Zfp422-mKO embryos at E17. Data are presented as mean ± SD. *n* (control) = 9, *n* (Zfp422-mKO) = 8. **f** Staining for MyHC and nuclei (DAPI) on E17 dorsal muscle cross sections. Mean myofiber CSA and the number of myofibers per 10,000 μm^2^ were counted. **g** mRNA expression of indicated genes in control and Zfp422-mKO E17 mouse dorsal muscle. **h** Myoblasts isolated from Zfp422-mKO E17 embryos and control were differentiated for 3 days, and stained for MyHC and nuclei. The differentiation index and fusion index were counted. Scale bars represent 50 μm in **b**, **c**, **f** and 100 μm in **h**. **p < 0.01, ***p < 0.001

To investigate Zfp422 expression profile during embryonic skeletal muscle development, we detected its mRNA expression in dorsal muscle of wild-type mouse embryos. It was highly expressed with a peak expression at E13 (Fig. 1a), indicating its involvement in embryonic myogenesis. At E17, its mRNA expression level is even higher than that of myosin heavy chain (MyHC) (Fig. 1a). Indeed, staining for Zfp422 and nuclei on cross section of dorsal muscle WT E17 embryos revealed that it was expressed in almost all nuclei in dorsal muscle (Fig. 1b, c), including progenitors (Pax7^+^), myoblasts (MyoD^+^), myofibers (MyHC^+^) as well as other nonmyogenic cells.

To examine its role in embryonic skeletal muscle development, mice carrying floxed Zfp422 alleles with loxP sites (Zfp422^fl/fl^) were crossbred with mice expressing Cre recombinase from endogenous Myf5 locus (Myf5^Cre/+^) to generate Zfp422^fl/fl^;Myf5^Cre/+^ (Zfp422-mKO) mouse embryos, in which exon 2 of the Zfp422 gene had been knocked out; Zfp422^+/+^;Myf5^Cre/+^ embryos were used as the control. As expected, the protein level of Zfp422 was dramatically reduced in dorsal muscle of Zfp422-mKO E17 embryo (Fig. 1d). As a result, the weight of Zfp422-mKO E17 embryos was significantly less compared with the control (Fig. 1e). Immunofluorescent staining for MyHC and calculation of mean myofiber cross-section area (CSA) showed that Zfp422-mKO embryos have smaller dorsal muscle myofibers (Fig. 1f). Although the number of myofibers per area did not reach statistical significance (Fig. 1f), the shape of dorsal muscles was not well organized compared with control (Fig. S1). Furthermore, the mRNA expression of *Myogenin, MRF4, TroponinT2, Myomaker, and MyHC* were significantly decreased in Zfp422-mKO embryos (Fig. 1g).

Further, primary myoblasts isolated from Zfp422^fl/fl^;Myf5^Cre/+^ and Zfp422^+/+^;Myf5^Cre/+^ embryos and were induced to differentiate after reaching confluence. As expected, immunofluorescent staining for MyHC proved that the differentiation of primary myoblasts isolated from Zfp422-mKO was impaired, both differentiation index and fusion index were dramatically decreased (Fig. 1h). Taken together, Zfp422 is essential for embryonic myogenesis.

### Zfp422 is required for muscle regeneration and satellite cell differentiation

**Fig. 2:**
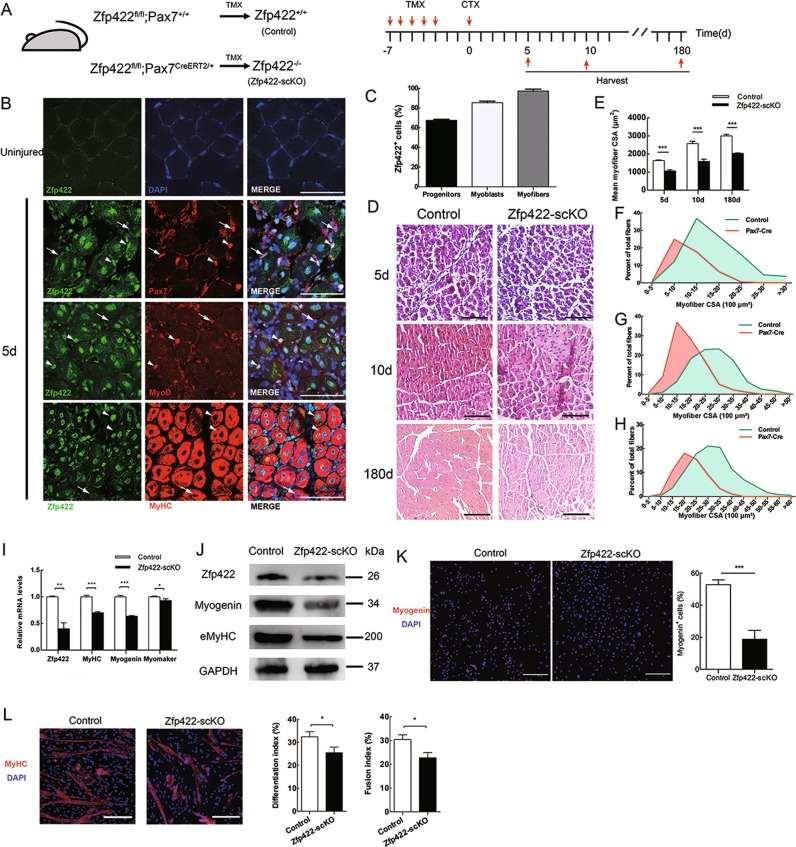
Zfp422 is required for muscle regeneration in vivo and satellite cell differentiation in vitro. **a** Schematic of TMX-induced Cre expression to knock out Zfp422 specifically in satellite cells followed by CTX injection-induced TA muscle regeneration. Zfp422fl/fl mice were crossbred with mice expressing Pax7-driven CreERT2 (Pax7CreERT2/+), a tamoxifen-inducible recombinase to generate Zfp422fl/fl;Pax7CreERT2/+ (Zfp422-scKO) mice, which enabled us to conditionally delete Zfp422 in satellite cells after tamoxifen (TMX) injection. 8-12-week-old Zfp422fl/fl;Pax7CreERT2/+ mice and Zfp422fl/fl;Pax7+/+ control mice were treated with TMX for 5 days, followed by CTX injection. Then TA muscles were harvested and analyzed 5, 10 and 180 days after injury. **b** Zfp422 expression during regeneration in Control adult mouse TA muscle. Arrowheads indicate the representative cells that express Zfp422, and arrows indicate the representative cells without Zfp422 expression. **c** Percentages of Zfp422-expressing cells at 5d during muscle regeneration for three muscle cell populations. **d** H&E staining on TA muscle sections at 5 d, 10 d and 180 d described in (**a**). **e** Mean CSA described in (**d**). **f**–**h** Size distributions of regenerating myofibers at 5 d (**f**), 10 d (G) and 180 d (**h**) were measured using ImageJ software. Data are presented as mean. **i** mRNA levels of indicated genes in regenerating TA on 5d. **j** Protein expression of indicated genes in regenerating TA on 5d. **k** Satellite cells isolated from Zfp422fl/fl;Pax7+/+ (Control) and Zfp422fl/fl;Pax7CreERT2/+ (Zfp422-scKO) EDL muscle were cultured in GM to proliferate for 4 days, then induced to differentiate in DM for 12 h. The percentage of Myogenin-expressing cells was calculated. **l** Satellite cells isolated from Control and Zfp422-scKO EDL muscle were differentiated for 3 days, and stained for MyHC and DAPI. The differentiation index and fusion index were counted. Data are presented as mean ± SD, *n* = 3 per group. Scale bars represent 50 μm in **b** and 100 μm in **d**, **k**, **l**. *p < 0.05, **p < 0.01, ***p < 0.001

We then studied the role of Zfp422 in muscle regeneration. First, we examined Zfp422 expression in TA muscle of Control mice during regeneration by co-staining with markers for different types of muscle cells as reported previously [45]. In uninjured TA muscle, Zfp422 expression was not detectable in nuclei (Fig. 2b, top panel). Five days after injury, it was expressed in 67.53 ± 0.91% of Pax7^+^ progenitors (satellite cells), 85.51 ± 1.51% of MyoD^+^ cells (activated myoblasts) and 97.92 ± 1.86% of centrally located nuclei of MyHC^+^ cells (regenerating myofibers) (Fig. 2b, c). These results indicate that Zfp422 is activated and gradually upregulated in differentiating myoblasts and regenerating myofibers.

After treatment illustrated in Fig. 2a, Zfp422 expression was decreased in TA muscle of Zfp422-scKO mice at 5 days (Fig. 2i, j). Hematoxylin and eosin (H&E) staining revealed less efficient regeneration characterized by smaller regenerated myofiber CSA in Zfp422-scKO mice at all time points (Fig. 2d–h). In addition, 5 days after injury, mRNA expression levels of *Myogenin*, *Myomaker*, and *MyHC* were significantly lower in regenerating muscles from Zfp422-scKO mice than control (Fig. 2i); similar to Myogenin, the protein level of embryonic MyHC (eMyHC), a marker of embryonic and regenerating myofibers, was also reduced (Fig. 2j). Together, these results indicate that Zfp422 is indispensable for muscle regeneration in vivo.

To further study the requirement for Zfp422 in satellite cell differentiation, satellite cells were isolated from the EDL of Zfp422-scKO and control mice that had been treated with TMX for continuous 5 days (Fig. S2). Satellite cells were first cultured in GM to proliferate, then induced for 12 h in DM followed by Myogenin staining. As a result, the percentage of Myogenin^+^ cells was significantly reduced in Zfp422-scKO satellite cells (Fig. 2k), indicating there is a differentiation defect in differentiating myoblasts without Zfp422. When induced to differentiate for 3 days, as expected, satellite cells from Zfp422-scKO mice formed less myotubes. Meanwhile, the differentiation index and fusion index were both decreased after differentiation (Fig. 2l). Collectively, these results indicate that Zfp422 is crucial during satellite cell differentiation and fusion in vitro.

### Zfp422 is required for myoblast differentiation

**Fig. 3:**
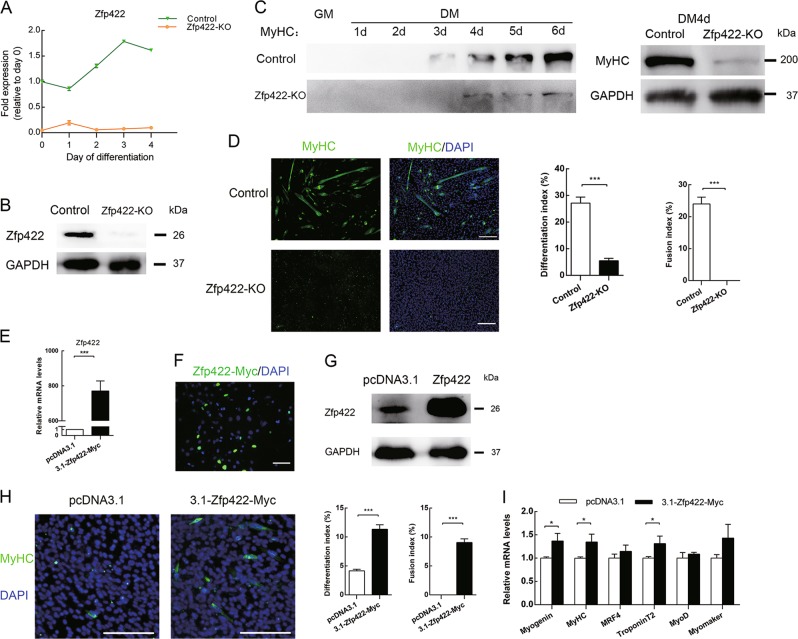
Zfp422 is required for myoblast differentiation. **a**, **b** Establishment of Zfp422 knockout (Zfp422-KO) C2C12 myoblasts using CRISPR-Cas9 system. Zfp422 mRNA (**a**) and protein (**b**) expression in Zfp422-KO and control C2C12 cells. **c** MyHC protein level in Zfp422-KO and control C2C12 cells at indicated time points. **d** Control and Zfp422-KO C2C12 were immunostained for MyHC at DM 4d. The differentiation index and fusion index were counted. Zfp422-KO myoblasts coundn't fuse to form myotube. **e** Zfp422 mRNA level in C2C12 cells after transfection with 3.1-Zfp422-Myc vector. **f** Immunouorescence of Myc 48 h after transfection with 3.1-Zfp422-Myc vector in C2C12. Scale bar represents 50 μm. **g** Protein level of Zfp422 48 h after transfection with 3.1-Zfp422-Myc vector in C2C12. **h** Immunostaining for MyHC at DM3d after control or Zfp422-expression vector transfection in Zfp422-KO cells. The differentiation index and fusion index were counted. **i** Expression of myogenic genes during DM in Zfp422-KO cells after control or Zfp422-expression vector transfection. Scale bar represents 100 μm. Data are presented as mean ± SD, *n* = 3 for each group. *p < 0.05, ***p < 0.001

To further clarify the requirement for Zfp422 in myoblast differentiation, we established a Zfp422 knockout (Zfp422-KO) C2C12 myoblast cell line (Figs. 3a, b, S3). In this cell line, Zfp422 expression was not detectable. Western blotting and immunofluorescence assays demonstrated that MyHC was reduced dramatically (Fig. 3c), and Zfp422-KO cells completely lost the ability to fuse and form myotube, and differentiation index decreased significantly (Fig. 3d). To determine whether exogenous expression of Zfp422 could rescue the inhibited differentiation, Zfp422 expression vector was successfully constructed (Fig. 3e–g) and transiently transfected into Zfp422-KO cells (Fig. 3h–i). As a result, myotubes formed successfully compared with transfecting empty vector, and both differentiation index and fusion index were increased (Fig. 3h). In addition, the expression of *MyHC*, *Myogenin*, and *TroponinT2* was rescued by exogenous expression of Zfp422 (Fig. 3i).

### Zfp422 influences the expression of myogenic genes

**Fig. 4:**
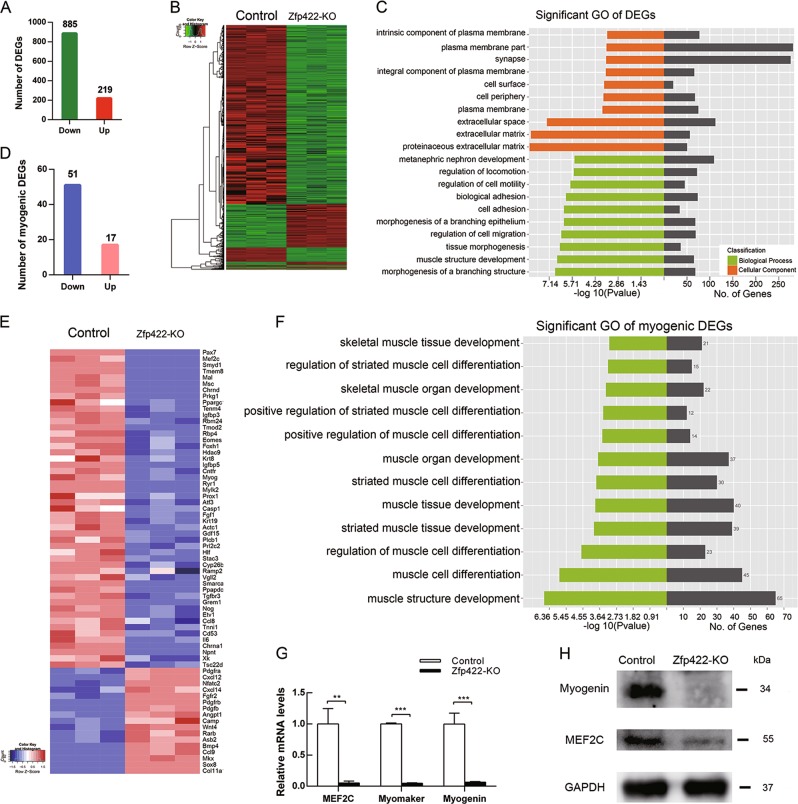
Identification of differentially expressed genes (DEGs) caused by Zfp422 knockout in C2C12 myoblasts. **a** The number of DEGs that downregulated or up-regulated. **b** Heat map of DEGs in (**a**). **c** Significant GO in biological processes and cellular component of DEGs. The lowest 10 *p* value of GO terms were presented. **d** The number of down-regulated or up-regulated DEG involved in muscle development. **e** Heat map of DEGs in (**d**). **f** Significant muscle GO biological processes of genes in (**e**). **g** The down regulation of mRNA levels of MEF2C, Myomaker and Myogenin was validated by qPCR. Data are presented as mean ± SD, *n* = 3 for each group. **p < 0.01, ***p < 0.001. **h** MEF2 and Myogenin protein levels in Zfp422-KO and control C2C12

RNA-seq analysis was performed to explore the genes influenced by Zfp422. Among 31,176 genes identified, 1104 genes with a fold change <0.5 or >2 showed statistical significance between the control and Zfp422-KO cell line. Eight hundred and eighty five of them were downregulated and 219 were upregulated (Fig. 4a, b). Gene ontology (GO) analysis revealed that these differentially expressed genes (DEGs) had significant enrichment in muscle development, cell migration, and movement related functions; cellular component had significant enrichment in extracellular matrix and membrane-associated components (Fig. 4c). Cell movement such as migration, adhesion, and fusion is dependent on cell–cell communication, in which extracellular matrix and membrane-associated proteins play vital roles, is also important in skeletal muscle development [1, 14]. These results might provide clues to find target genes for Zfp422.

In line with the reduced myotube formation, there are 68 genes out of these 1104 DEGs were involved in myogenesis, with 51 of them downregulated and 17 upregulated (Fig. 4d, e). These genes had significant enrichment of functional annotations for multiple muscle-related biological processes (Fig. 4f). The RNA-seq results about *myocyte enhancer factor-2* (*MEF2C*), *Myomaker* (*Tmem8*), and *Myogenin*, which are key proteins implicated in myoblast differentiation [3, 21, 28], were validated by qPCR (Fig. 4g). MEF2 and Myogenin expression was also reduced (Fig. 4h). Collectively, Zfp422 is required for induction of myogenic genes.

### EphA7 is a downstream target of Zfp422

**Fig. 5:**
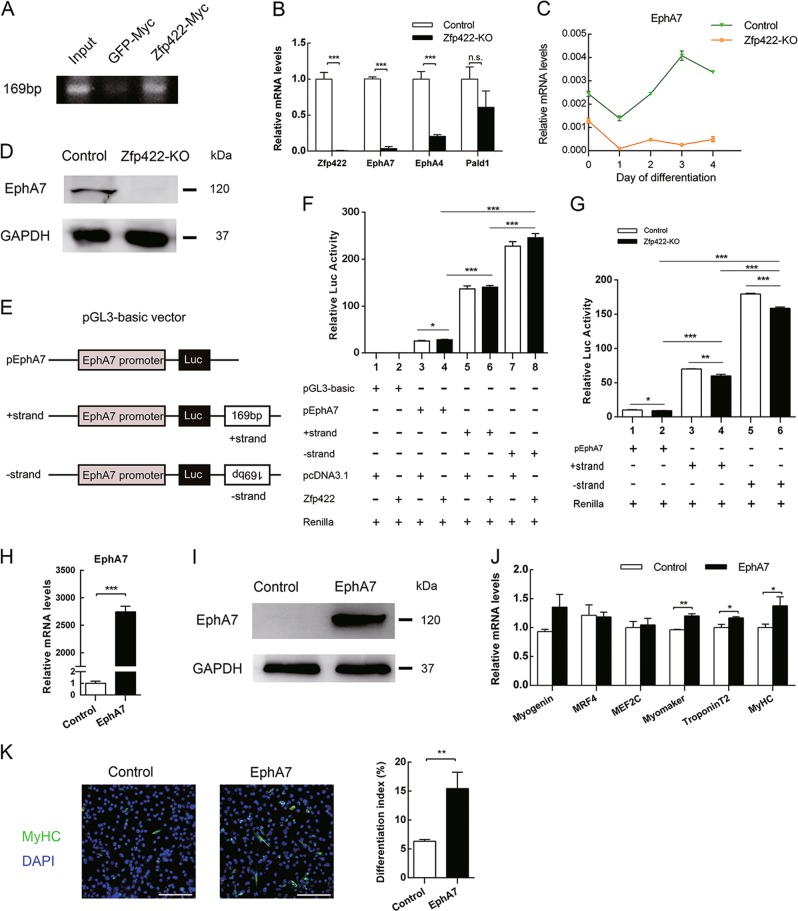
EphA7 is a downstream target of Zfp422 and rescues the differentiation of Zfp422-KO C2C12 myoblasts. **a** C2C12 cells were transfected with 3.1-Zfp422-Myc vector to introduce exogenous Myc-tagged Zfp422 expression, 3.1-GFP-Myc vector acted as a control. Then ChIP-seq was performed using Myc antibody. PCR validated the 169bp sequence bound by Zfp422 from the ChIP-seq results. **b**, **c** EphA7, EphA4 and Pald1 mRNA expression in Zfp422-KO C2C12 cells. **d** EphA7 protein expression in Zfp422-KO and control C2C12 cells. **e** Schematic of reconstructed pGL3-baisc luciferase reporter vector. **f** C2C12 cells were transiently transfected with Zfp422 expression vector or empty vector, together with other indicated luciferase reporter vectors, then measured dual luciferase activity 48 h later. **g** Zfp422-KO and control C2C12 cells were transiently transfected with the indicated luciferase reporter vectors, then harvested and measured dual luciferase activity 48 h later. **h**, **i** EphA7 mRNA expression (**h**) and protein level (**i**) in Zfp422-KO C2C12 cells after transfection with 3.1-EphA7 vector. **j** The expression changes of genes related to myoblast differentiation after EphA7 overexpression in Zfp422-KO C2C12 cells. **k** Immunostaining for MyHC at DM3d after control or EphA7-expression vector transfection in Zfp422-KO cells. Differentiation index was counted. Data are presented as mean ± SD, *n* = 3 for each group. *p < 0.05, **p < 0.01, ***p < 0.001. n.s., no significance

To investigate the direct target genes regulated by Zfp422, ChIP-seq was performed. Among 113 peaks bound by Zfp422-Myc, a site (169 bp) that located at 636 kb upstream of *EphA7* transcription start site (TSS) caught our attention (Table S1), as the expression *EphA7* as well as *EphA4*, was revealed to be significantly inhibited in Zfp422-KO C2C12 cells by RNA-seq (Table S2). This binding was further confirmed using ChIP-PCR (Fig. 5a). The RNA-seq results about *EphA7* and *EphA4* were also validated by qPCR (Fig. 5b). *Pald1* was another gene that bound by Zfp422-Myc, but its expression was not changed after Zfp422 knockout (Fig. 5b). For the above reasons, we then focused on EphA7 next. *EphA7* expression was dramatically reduced in Zfp422-KO myoblasts (Fig. 5c, d). What’s more, the Pearson correlation coefficient of *Zfp422* and *EphA7* mRNA expression was over 0.986 (Figure S4), indicating high correlation between them.

As the binding site of Zfp422 is far away from *EphA7* TSS, we speculate that it may be an enhancer sequence for *EphA7*. To verify this speculation, *EphA7* promoter was cloned into a pGL3-basic luciferase reporter vector, or together with the + strand or -strand of binding sequence (Fig. 5e). Reporter vectors were co-transfected into C2C12 cells with empty or plasmids expressing Zfp422 in various combinations as indicated in Fig. 5f. As a result, reporter vectors containing the binding site strongly increased the activity of *EphA7* promoter, and the -strand was more efficient (Fig. 5f). The overexpression of Zfp422 tended to elevate the transcriptional activity, but the differences did not reach statistical significance, possibly because of a maximal response to endogenous Zfp422 activity (Fig. 5f). Then we performed another reporter assay in Zfp422-KO and control C2C12 cells (Fig. 5g). As expected, the activity of *EphA7* promoter was much weaker in Zfp422-KO cells (Fig. 5g). These results suggest that this binding site is an enhancer of *EphA7*, and Zfp422 promotes the expression of EphA7 by binding to it directly.

Further, to determine whether exogenous expression of EphA7 could rescue the compromised differentiation and fusion of Zfp422-KO cells, EphA7 expression vector was transiently transfected into Zfp422-KO myoblasts (Fig. 5h, i). As a result, the mRNA expression levels of *Myogenin*, *Myomaker*, *MyHC* and *TroponinT2* were partially rescued (Fig. 5j). Furthermore, differentiation index increased significantly (Fig. 5k). Unfortunately, myotube formation wasn’t promoted obviously, indicating EphA7 was unable to rescue the inhibited myoblast fusion caused by *Zfp422* knockout. This may due to the fact that it’s not the only target gene of Zfp422 during myogenesis.

### EphA7 is necessary for myoblast differentiation

**Fig. 6:**
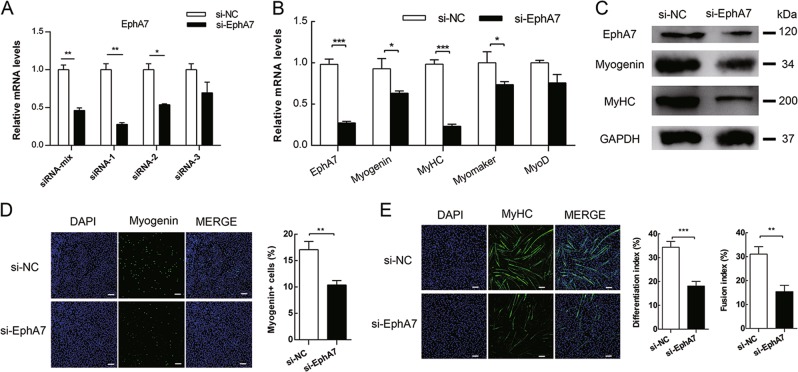
EphA7 is necessary in myoblast differentiation. C2C12 cells were transfected with negative control siRNA (si-NC) or EphA7 siRNA (si-EphA7), then induced to differentiate 2d later. **a** Knockdown efficiency of different EphA7 siRNA. **b**, **c** The mRNA levels (**b**) and protein expression (**c**) of indicated genes in C2C12 after EphA7 mRNA interference. **d** Percentage of Myogenin-positive cells in C2C12 cells after EphA7 knockdown at DM1d. **e** MyHC staining in C2C12 cells after si-EphA7 transfection at DM3d. Differentiation index and fusion index were calculated. Scale bars represent 100 μm. Data are presented as mean ± SD, *n* = 3 per group. *p < 0.05, **p < 0.01, ***p < 0.001

To further investigate the role EphA7 plays in myoblast differentiation and fusion, endogenous EphA7 was knocked down by siRNA (Fig. 6a). Knockdown of EphA7 significantly reduced the mRNA levels of *Myogenin*, *MyHC*, and *Myomaker* (Fig. 6b). Similarly, Myogenin and MyHC were also decreased (Fig. 6c). Immunofluorescence assay showed that knockdown of EphA7 in C2C12 cells reduced the percentage of cells expressing Myogenin at DM 1d (Fig. 6d), and MyHC expression was inhibited, which resulted in decrease of differentiation index and fusion index (Fig. 6e). Therefore, EphA7 is necessary for myoblast differentiation and fusion in vitro.

### EphA7 is required for proper apoptosis during myogenic differentiation

Previous studies showed that EphA7 is involved in cell apoptosis [42, 46, 47], which has influence upon muscle development [22]. In addition, the number of Zfp422-KO cells at DM4d was apparently more than that of control cells (Fig. 3d), although Zfp422 knockdown did not affect C2C12 cell proliferation (Fig. S6D). This could be due to that less Zfp422-KO cells underwent cell death after induction of differentiation. Therefore, we next studied whether EphA7 affected apoptosis during myoblasts differentiation.

**Fig. 7:**
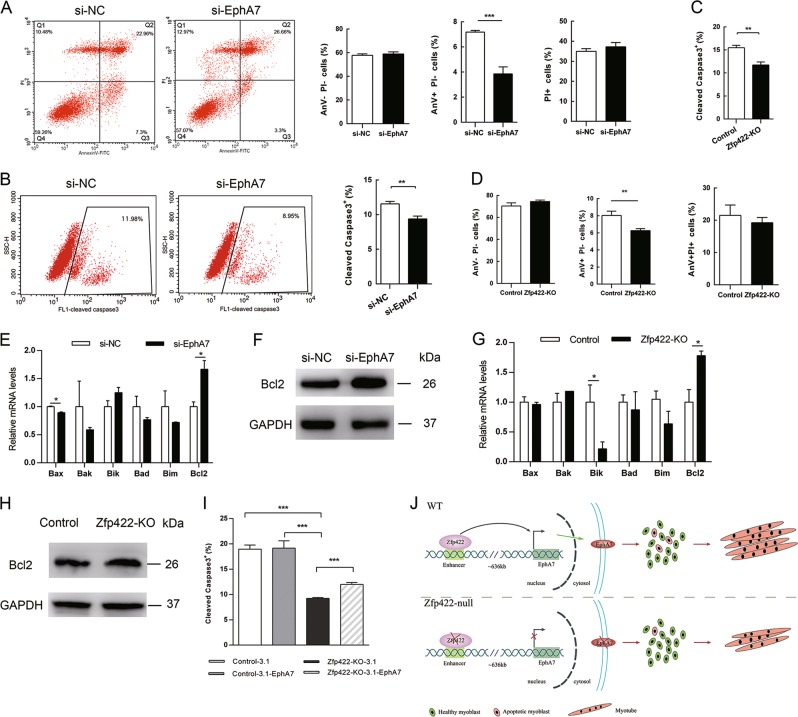
Zfp422 and EphA7 are required for proper apoptosis in myoblasts differentiation. **a** Fluorescence-activated cell sorting analysis of cell death. C2C12 cells were transfected with si-NC or si-EphA7, and induced to differentiate 48 h later. After differentiation for 24 h, cells were double stained with PI and AnV. Percentage of viable cells (AnV-PI-), apoptotic cells (AnV+PI-), and necrotic cells (PI+) were calculated. **b** C2C12 cells were transfected with si-NC or si-EphA7, and induced to differentiate 48 h later. After 24 h, cleaved caspase-3 was stained and analyzed by flow cytometry. **c** Percentage of cleaved caspase-3 positive cells in Zfp422-KO and control C2C12 cells at DM 1d. **d** Percentage of viable cells, apoptotic cells, and necrotic cells of Zfp422-KO and control C2C12 cells at DM 1d. **e** mRNA levels of Bcl2 family apoptotic genes in C2C12 after EphA7 mRNA interference at DM1d. **f** Bcl2 protein level in C2C12 after EphA7 mRNA interference at DM1d. **g** mRNA levels of Bcl2 family apoptotic genes in control and Zfp422-KO C2C12 cells at DM1d. **h** Bcl2 protein expression in control and Zfp422-KO C2C12 cells at DM1d. **i** Percentage of cleaved caspase-3 positive cells in control and Zfp422-KO C2C12 cells at DM 1d after transfecting empty or EphA7 expression vector. **j** Schematic of Zfp422 promotes myogenesis via enhancing EphA7 expression to maintain proper fraction of apoptosis in myoblasts, and eventually results in more muscle formation. Scale bar represents 50 μm. Data are presented as mean ± SD, *n* = 3 per group. *p < 0.05, **p < 0.01, ***p < 0.001

Double staining with AnV and PI analysis showed significant decrease of AnV^+^PI^−^ population (apoptotic cells) at DM 1d in cells transfected with si-EphA7 (Fig. 7a). Simultaneously, the percentage of active caspase-3 positive cells presented a significant decrease at this time (Fig. 7b). These results confirm the hypothesis that EphA7 positively regulates apoptosis in early differentiation of C2C12 cells. Similarly, the proportion of both AnV^+^PI^−^ population and active caspase-3 positive cells showed significant decrease in Zfp422-KO C2C12 cells compared with that in control cells (Figs. 7c, d, S6A–B). Moreover, the proportion of cleaved caspase-3 positive cells was partially rescued by forced expression of EphA7 (Figs. 7i, S6C).

Besides, myogenic differentiation of C2C12 cells is accompanied by enhanced apoptosis in a fraction of cells, and Bcl2 family of apoptosis regulators has been reported to be expressed and function in this process [48, 49]. Therefore, the expression changes of Bcl2 family genes were examined after EphA7 knockdown in differentiating C2C12 cells. The results showed that expression of anti-apoptotic gene Bcl2 was elevated, whereas expression of pro-apoptotic gene Bax was inhibited (Fig. 7e, f). Similarly, in differentiating Zfp422-KO C2C12 cells, Bcl2 expression was increased, while pro-apoptotic gene Bik expression was inhibited (Fig. 7g, h). Altogether, these results support the idea that EphA7 induces appropriate amount of cell apoptosis in muscle cells.

## Discussion

In this study, we identified that a membrane receptor gene *EphA7* is a direct downstream target gene of Zfp422. ChIP-seq analysis found a Zfp422 binding site located at 636 kb upstream of *EphA7* TSS, which was an enhancer of *EphA7* confirmed by luciferase reporter assay (Fig. 5e–g). *EphA7* expression was highly correlated to *Zfp422* expression during embryonic and postnatal muscle development in vivo and during myoblast differentiation in vitro, and was dramatically inhibited in Zfp422-KO myoblasts (Figs. 5c, S4). Furthermore, EphA7 was proved to be required in myoblast differentiation and fusion to form myotubes (Fig. 6).

EphA7 has a truncated form, which is a secreted protein [50]. The secreted EphA7 promotes somatic cell reprogramming by inducing ERK activity reduction [51]. Another study in prostate tumor growth and progression demonstrated that ligand-dependent EphA7 containing tyrosine site induced prostate tumor cell apoptosis, but the truncated secreted form EphA7 did not have this effect [42]. In the present study, forced expression of full length EphA7 rescues the differentiation in Zfp422-KO myoblasts partially, while secreted EphA7 cannot (Figs. 5h–k, S5C). Besides, addition of secreted EphA7 has no influence on myoblast differentiation (Fig. S5A–B). These studies indicate that there is functional diversity between the two forms of EphA7.

Apoptosis is the process of programmed cell death, and appropriate apoptosis is necessary in multicellular organisms. Excessive apoptosis causes atrophy including skeletal muscle atrophy [52], whereas an insufficient amount results in uncontrolled cell proliferation, and leads to many types of cancer [53]. Therefore, there should be delicate balance between cell survival and death signal. During the process of myogenic differentiation of C2C12 cells, a fraction of myoblasts within the population undergoes apoptosis, which is required for proper myotube formation [22]. EphA7 together with its ligand ephrinA5 is proved to induce HEK293 cell apoptosis via TNFR1 [46] and caspase-8 [47]. These studies suggest the possibility of EphA7 involving in myoblast apoptosis. Actually, EphA7 is proved to be required for proper apoptosis in myogenic differentiation in our study (Fig. 7). Importantly, how does EphA7 regulate the subset of myoblasts that become apoptotic? Previous studies in brain development demonstrated that ephrinA5/EphA7 signaling promoted apoptosis to control brain size, which revealed the critical role of ephrinA5 played in EphA7-mediated apoptosis [54, 55]. Meanwhile, another study proved that ligand-dependent EphA7 but not the truncated secreted form of EphA7 induced prostate cancer cell apoptosis; and tyrosine 791 phosphorylation of EphA7, which is required in prostate cancer cell apoptosis, is dependent on the stimulation of ephrinA5 ligand [42]. Therefore, activity and function of EphA7 in regulating myoblast apoptosis are probably dependent upon not only expression of its ligand ephrinA5 in neighboring cells, but also its phosphorylation level promoted by ephrinA5. EphA7-expressing cells probably will not be apoptotic without receiving apoptotic signaling from neighboring ephirnA5 positive cells by cell–cell contact. On the other hand, consistent with the previous study [42], our work showed that knockdown of EphA7 in myoblasts decreased the caspase-3 activity, elevated the protein expression level of Bcl2 (Fig. 7b, f). However, the molecular mechanism underlying how EphA7 regulates apoptosis in myoblasts remains to be further investigated.

The molecular mechanism by which apoptosis promotes myoblast fusion has been explained previously [22]. Transient externalization of phosphatidylserine is necessary for myoblast fusion [17]. Apoptotic cells also expose phosphatidylserine, which is a ligand for BAI1. BAI1 then enhances myoblast fusion by signaling via ELMO/Dock180/Rac1 proteins. Coincidentally, EphA7 is reported to enhance HSPC maintenance, migration, and adhesion through Rac1 activation [41]. Rac1 is also essential for myoblast fusion in mouse [56]. Thus, EphA7 is likely to positively regulate myoblast fusion. In fact, our work proved the requirement of EphA7 in myoblast differentiation and fusion to form myotubes in vitro (Fig. 6). As a downstream target of Zfp422, EphA7 overexpression in Zfp422-KO C2C12 cells partially rescued the compromised differentiation (Fig. 5j, k) and the amount of apoptotic cells (Fig. 7i). However, myoblast fusion was not rescued by exogenous EphA7 expression (Fig. 5k). That is, the partially rescued apoptotic Zfp422-KO C2C12 cells by overexpressing EphA7 did not promote myoblast fusion well, which is different from previous study that adding back apoptotic C2C12 myoblasts restored the impaired fusion caused by blocking apoptosis [22]. On one hand, this failed rescue may reflect the requirement of signaling from other target genes of Zfp422 to cooperate with EphA7 in myoblast fusion, since EphA7 is not the only target gene of Zfp422. On the other hand, it may due to the differences between endogenous and exogenous EphA7, such as the expression levels and expression timing as well as posttranslational regulation of EphA7, which may result in functional differences.

In summary, as illustrated in Fig. 7f, Zfp422 promotes myogenesis via enhancing EphA7 expression to maintain proper fraction of apoptosis in myoblasts. Zfp422 binds to an enhancer located at upstream of *EphA7* and promotes its expression. EphA7 is essential for an appropriate apoptotic proportion of myoblasts during early myogenic differentiation. This study reveals the regulation of membrane receptor EphA7 by a zinc finger transcription factor Zfp422, and their importance in muscle development. However, the molecular mechanism by which EphA7 induces apoptosis in myoblasts and the specific ephrin ligands that EphA7 recognizes during this process are not clear, remain to be further studied.

## Supplementary information

Supplementary information

## Data Availability

The RNA-seq raw data from this study have been deposited in NCBI Sequence Read Archive with accession number PRJNA556331 (https://www.ncbi.nlm.nih.gov/sra/).
