# Supplementary material for: Zfp422 promotes skeletal muscle differentiation by regulating EphA7 to induce appropriate myoblast apoptosis
Source: Cell Death Differ. 2019 Nov 4;27(5):1644–59. doi: 10.1038/s41418-019-0448-9 (PMC7206035; doi:10.1038/s41418-019-0448-9)
Supplement: Supplementary file 1 — Supplementary information [file 41418_2019_448_MOESM1_ESM.docx]

**Supplementary information**

**Zfp422 promotes skeletal muscle differentiation by regulating EphA7 to induce appropriate myoblast apoptosis**

Running title: Zfp422 promotes myogenesis by regulating EphA7

Yaping Nie#, Shufang Cai#, Renqiang Yuan, Suying Ding, Xumeng Zhang, Luxi Chen, Yaosheng Chen, Delin Mo*

SUPPLEMENTARY FILE

Supplemental Figures


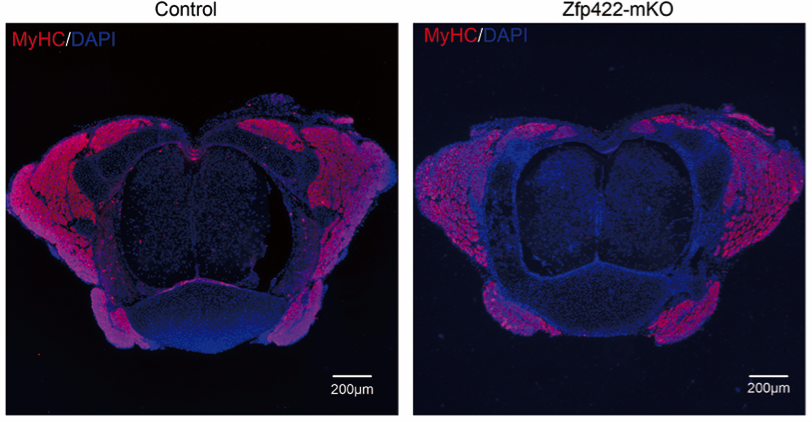


Figure S1. Representative cross section immunostaining images showing that in comparison with control (*Zfp422^+/+^;Myf5^Cre/+^*), dorsal muscles from E17 Zfp422-mKO (*Zfp422^fl/fl^;Myf5^Cre/+^*) mouse embryo were not well organized in shape.


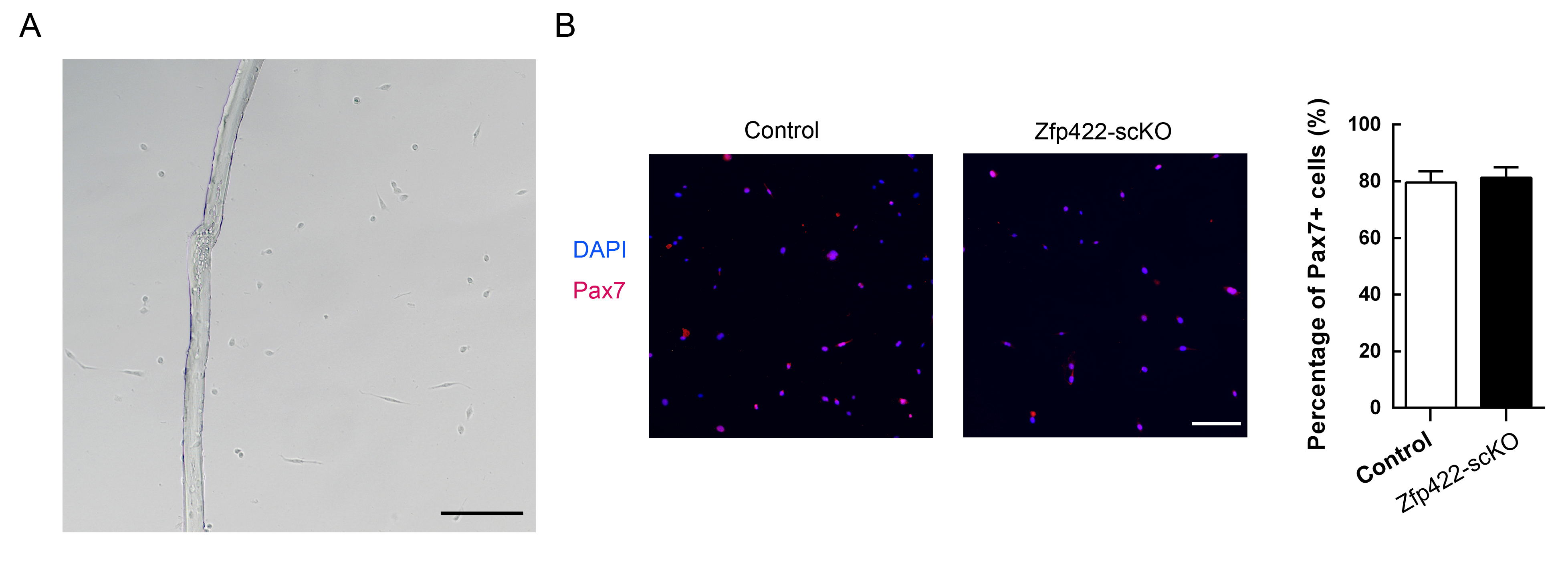


Figure S2. Identification of the isolated satellite cells. (A) Representative image of isolated live single myofiber with satellite cells in culture for 3d, some satellite cells migrate from the myofiber. (B) Myofiber was removed and the remained cells were stained for Pax7 and nuclei. Percentage of Pax7^+^ cells was counted. Scale bars represent 100 μm. Data are presented as mean ± SD, n=3 per group.


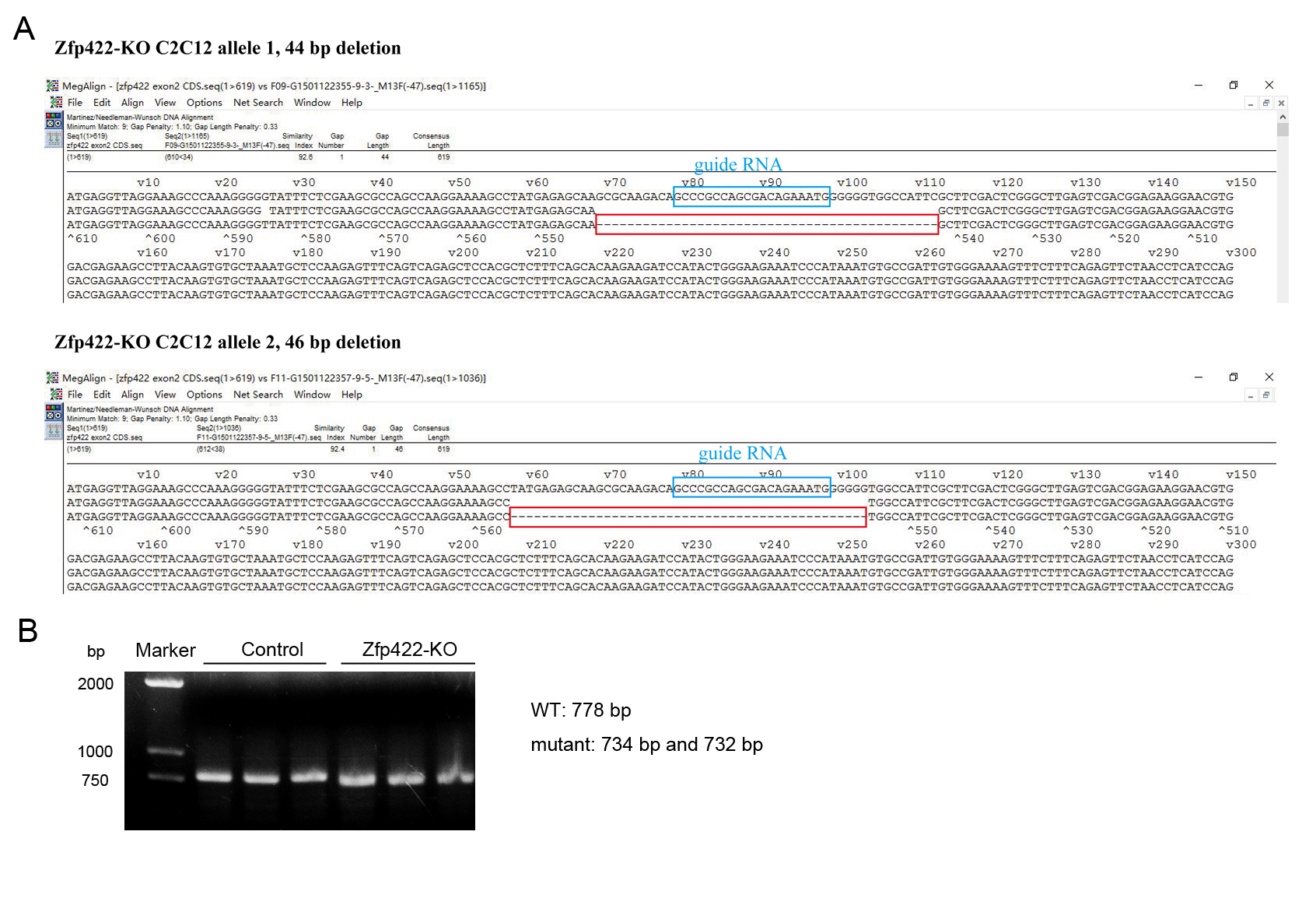
Figure S3. Confirmation of deletion in the *Zfp422* gene locus. The genotype of Zfp422-KO C2C12 was *Zfp422*^-44/-46^ compared to the wild type control. (A) The position of guide RNA is indicated by the blue boxes. The red boxes indicate deleted sequences in the alleles. This cell line has a 44-bp deletion in one allele, and a 46-bp deletion in the other allele. (B) PCR confirmed the deletions.


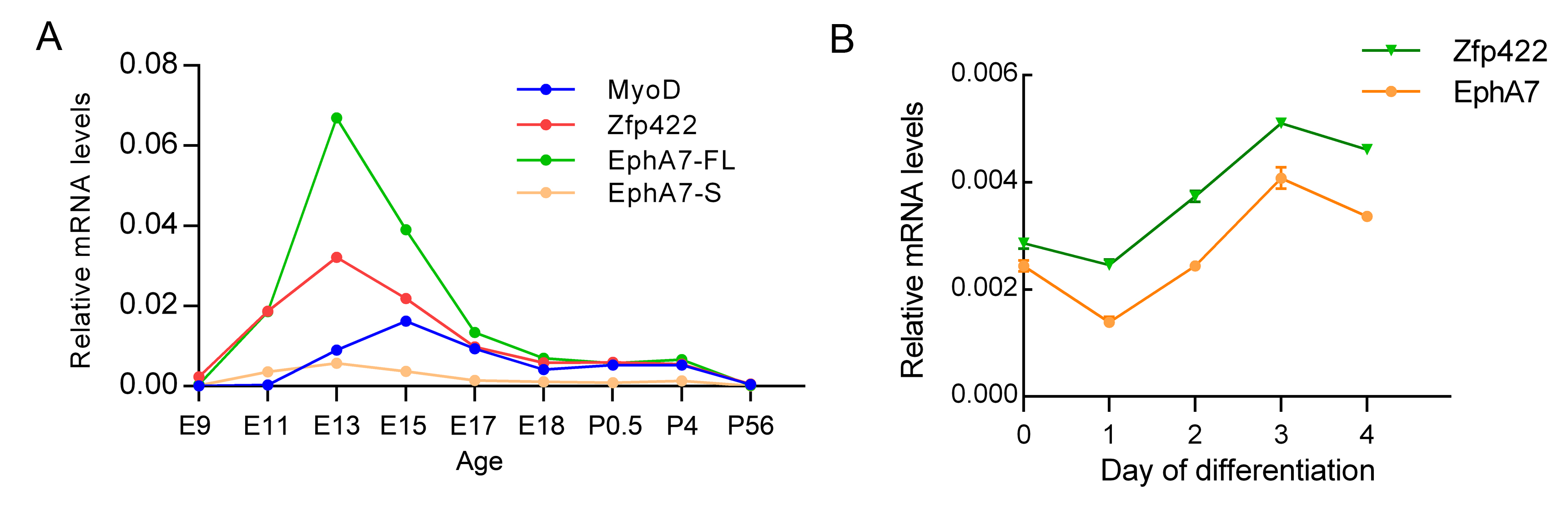
Figure S4. The correlation between *Zfp422* and *EphA7* expression. (A) Expression for *MyoD*, *Zfp422*, full length *EphA7* (*EphA7-FL*) and secreted *EphA7* (*EphA7-S*) of mouse dorsal muscle during embryonic and postnatal development. (B) Expression of *Zfp422* and *EphA7* during C2C12 differentiation. Data are presented as a single value (A) or mean ± SD from three biological replicates in the same experiment (B).


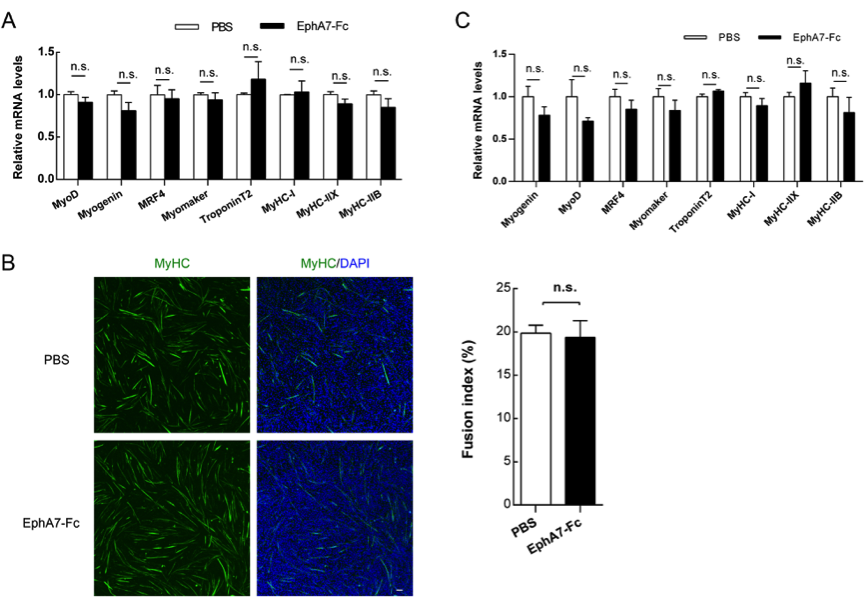


Figure S5. EphA7-Fc has no effect on C2C12 differentiation and can't rescue the differentiation of Zfp422-KO C2C12 cells. (A) EPHA7-Fc (5 μg/ml) was added to the medium of cultured C2C12 cells from GM to DM3d, mRNA levels of indicated genes at DM3d were measured by qPCR. (B) Immunoﬂuorescence for MyHC with or without EphA7-Fc addition at DM3d. (C) EPHA7-Fc was added to the medium of cultured Zfp422-KO C2C12 cells from GM to DM3d, mRNA levels of indicated genes at DM3d were measured by qPCR. Scale bar represents 100 μm. Data are presented as mean ± SD, n=3 per group. n.s., no significance.


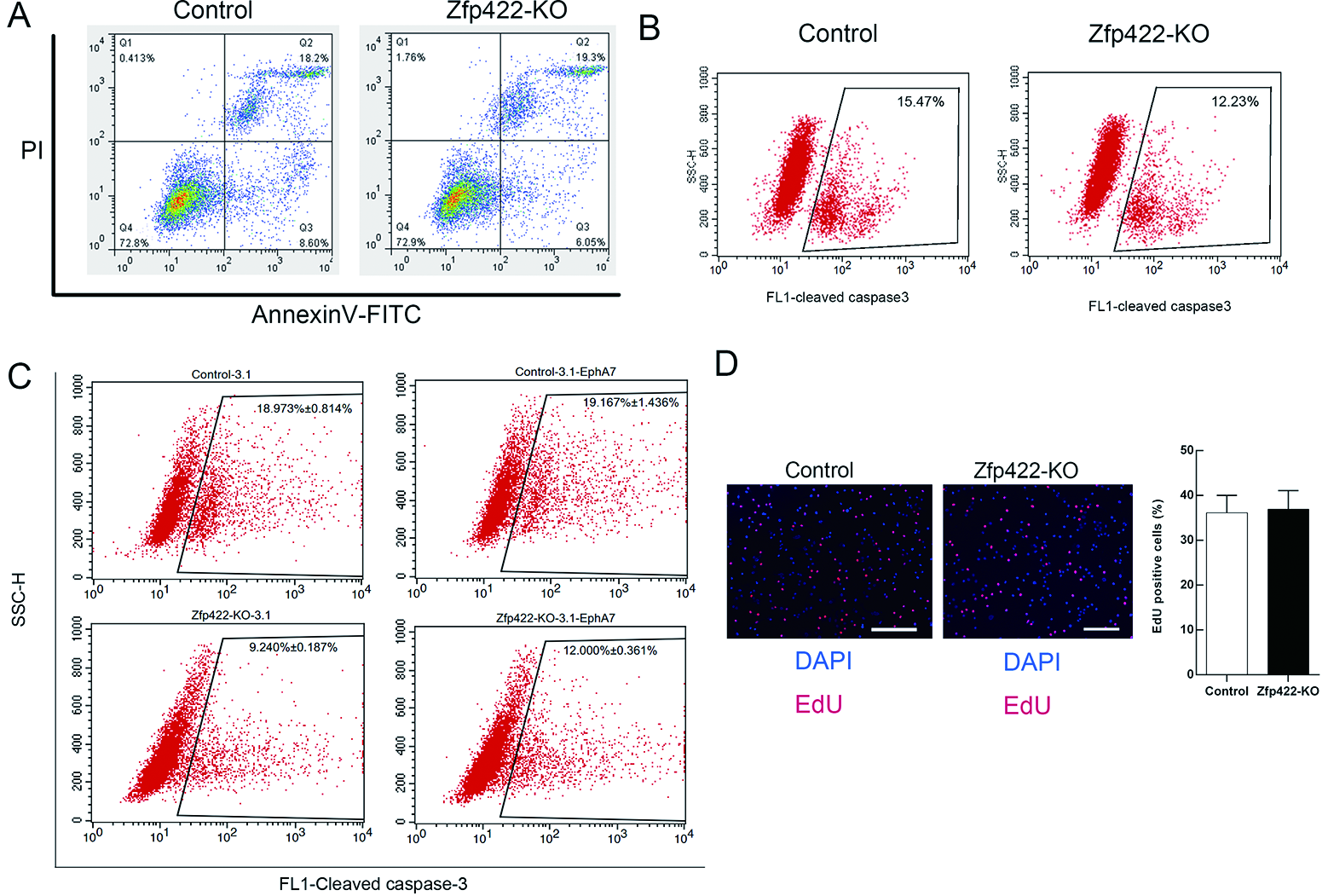


Figure S6. Apoptosis is inhibited in Zfp422-KO myoblasts, while their proliferation ability is not influenced. (A-B) Fluorescence-activated cell sorting analysis of PI and AnV double staining (A) and cleaved caspase-3 staining (B) on control and Zfp422-KO C2C12 cells at DM 1d. (C) Fluorescence-activated cell sorting analysis of cleaved caspase-3 staining on control and Zfp422-KO C2C12 cells at DM 1d after transfecting empty vector or EphA7 expression vector. (D) EdU staining on proliferating control and Zfp422-KO C2C12 cells. Scale bar = 100μm.


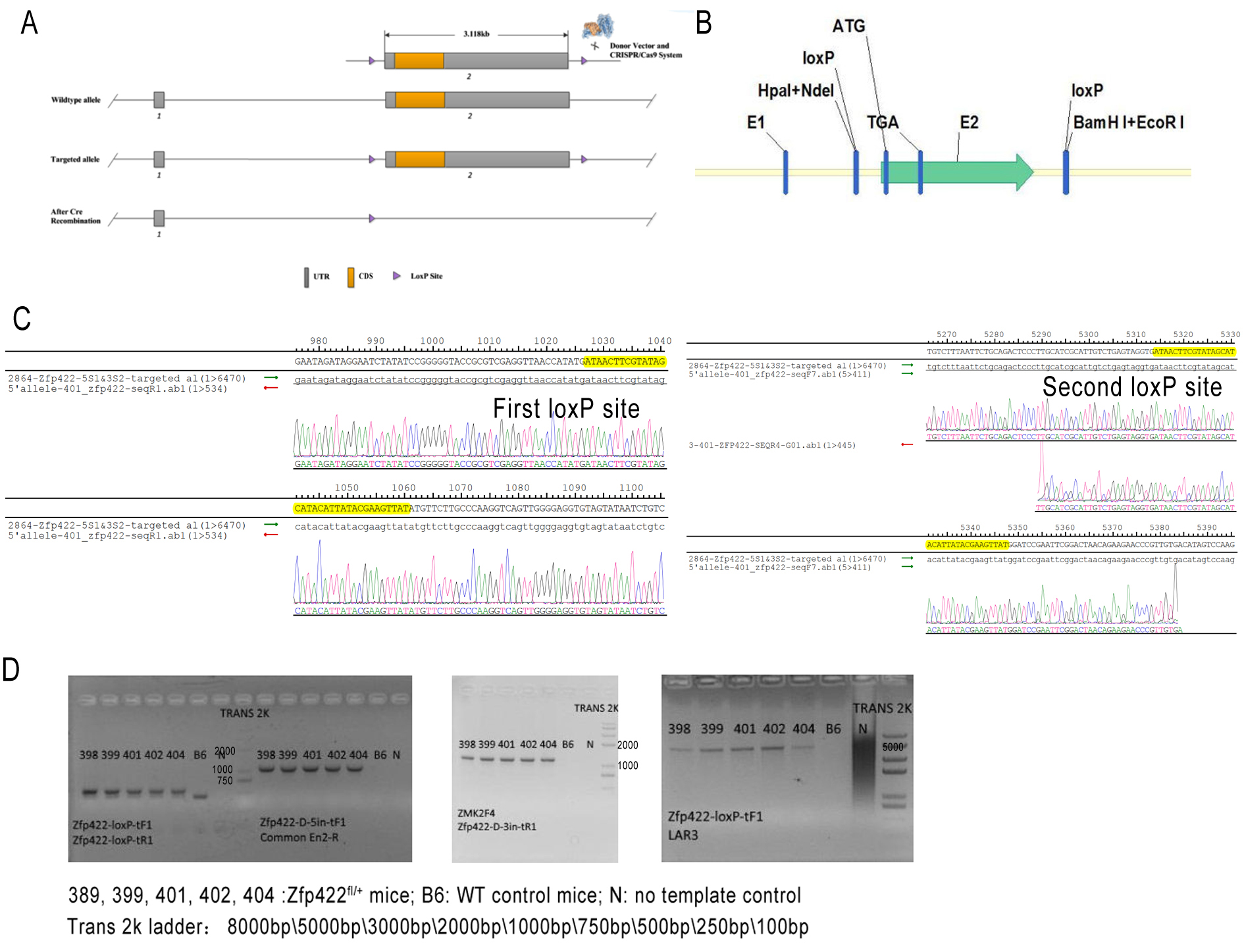


Figure S7. The genotype of Zfp422^fl/+^ mice was confirmed by PCR and sequencing of tail DNA. (A) Schematic of strategy for making *Zfp422^fl/+^* mice via CRISPR/Cas9 system. (B) Schematic for donor vector construction. (C) DNA sequencing results, the inserted loxP sites are highlighted in yellow. (D) PCR confirmed sequencing results.

Supplemental Tables

**Table S1.** Zfp422-Myc binding DNA sites (excluding sites in close proximity with GFP-Myc binding sites)

| PeakID | Chr & strand | Start | End | Annotation | Distance to TSS | Nearest Gene Name |
| --- | --- | --- | --- | --- | --- | --- |
| chr6-1 | chr6+ | 116626471 | 116626639 | exon 2 | 2146 | Zfp422 |
| chr10-3 | chr10+ | 58225191 | 58225359 | Intergenic | 7400 | Dux |
| chr10-2 | chr10+ | 58223910 | 58224078 | Intergenic | 8681 | Dux |
| chr8-4 | chr8+ | 20597462 | 20597630 | Intergenic | -17381 | Gm21119 |
| chr8-3 | chr8+ | 19707430 | 19707598 | Intergenic | -22062 | Gm21119 |
| chr5-2 | chr5+ | 15004611 | 15004779 | Intergenic | -25796 | Gm10354 |
| chr1-11 | chr1+ | 48881500 | 48881668 | Intergenic | -1414459 | Mir6350 |
| chr7-1 | chr7+ | 12010702 | 12010870 | Intergenic | -30513 | Vmn1r77 |
| chr8-1 | chr8+ | 20379395 | 20379563 | Intergenic | -16277 | Gm15319 |
| chr12-5 | chr12+ | 10693780 | 10693948 | Intergenic | 247476 | Gm38407 |
| chr15-4 | chr15+ | 101958735 | 101958903 | Intergenic | -4532 | Krt78 |
| chr10-5 | chr10+ | 58239433 | 58239601 | Intergenic | -2857 | Gm4981 |
| chr4-3 | **chr4+** | **28176897** | **28177065** | **Intergenic** | **-636150** | **Epha7** |
| chr5-4 | chr5+ | 15040085 | 15040253 | Intergenic | -7162 | Gm17019 |
| chr1-8 | chr1+ | 26686871 | 26687039 | intron 1 | 505 | 4931408C20Rik |
| chr10-4 | chr10+ | 61373980 | 61374148 | intron1 | 9459 | Pald1 |
| chr1-6 | chr1+ | 85090975 | 85091143 | intron 5 | 18794 | A530032D15Rik |
| chr1-5 | chr1+ | 26687446 | 26687614 | promoter-TSS | -70 | 4931408C20Rik |
| chr1-3 | chr1+ | 26688110 | 26688278 | promoter-TSS | -734 | 4931408C20Rik |
| chr5-5 | chr5+ | 15680658 | 15680826 | promoter-TSS | 32 | Speer4cos |
| chr8-2 | chr8+ | 107551292 | 107551460 | TTS | 132 | Mir140 |
| chr11-95 | chr11+ | 77077923 | 77078091 | TTS | 165 | Mir423 |

**Table S2.** RNA-seq results of Zfp422-Myc binding site-related genes and *Epha4*

| Gene | Control | Zfp422-KO | Fold Change | p-value |
| --- | --- | --- | --- | --- |
| *Epha7* | **101.5721786** | **1.287846691** | **0.01267913** | **5.31E-11** |
| *Dux* | 1.379738049 | 0 | 0 | 0.240574887 |
| *Krt78* | 0.703623921 | 0.361722263 | 0.51408466 | 0.9512873 |
| *Mir140* | 3.83219816 | 3.012568085 | 0.78612012 | 0.875114416 |
| *Mir423* | 1.38643038 | 0.723444527 | 0.52180372 | 0.717960579 |
| *Pald1* | 800.5805957 | 832.7963909 | 1.04024054 | 0.54031027 |
| *Zfp422* | 784.2234899 | 55.81232527 | 0.0711689 | 1.89E-123 |
| *Gm4981* | 0.696561356 | 0.266198989 | 0.38216158 | 0.94958904 |
| *Gm17019* | 1.373045718 | 0 | 0 | 0.240919415 |
| *Gm15319* | 0 | 0.361722263 | Inf | 1 |
| *Gm21119* | 0 | 0 | NA | NA |
| *Vmn1r77* | 0 | 0 | NA | NA |
| *A530032D15Rik* | 0.341436123 | 0.330062985 | 0.966690291 | 1 |
| *4931408C20Rik* | 0 | 0 | NA | NA |
| *4931408C20Rik* | 0 | 0 | NA | NA |
| *Epha4* | 1123.704046 | 516.8175297 | 0.459923173 | 1.29E-39 |

Inf: infinite; NA: not available

**Table S3**. Primers for qPCR (5’ to 3’)

| Gene name | sequence |
| --- | --- |
| *Myogenin* | F:GCAATGCACTGGAGTTCG  R:ACGATGGACGTAAGGGAGTG |
| *MyHC* | F:AGCTTGAAAACGAGGTGGAA  R: CCTCCTCAGCCTGTCTCTTG |
| *MyoD* | F: GCCTGAGCAAAGTGAATGAG  R: GCAGACCTTCGATGTAGCG |
| *MRF4* | F: CTACATTGAGCGTCTACAGGACC  R: CTGAAGACTGCTGGAGGCTG |
| *Zfp422* | F: ACAGCCCGCCAGCGACAGAAAT  R: ACTCTTGGAGCATTTAGCACAC |
| *EphA7*  *(EphA7-FL)* | F:CCGAGGAAGAGGCAGAAAA  R: TGGACAACGAGAACACTGGA |
| *EphA7-S* | F: TGCCCATGCAAATTACACTTTCG  R: GTAAGATCCCCCTGCACTTACC |
| *EphA4* | F: TCGTGGTCATTCTCATTG  R: TCTCTTCATCTGCTTCTTG |
| *Pald1* | F: AAGGAGGATGGTGGAGGAGG  R: GGCTGAAACTGAGGGCAAAG |
| *Myomaker* | F: ATCGCTACCAAGAGGCGTT  R: CACAGCACAGACAAACCAGG |
| *TroponinT2* | F: CAGCAGCGTATTCGCAATGA  R: TCTGGATGTACCCTCCAAAGTG |
| *MEF2C* | F: ATCCCGATGCAGACGATTCAG  R: AACAGCACACAATCTTTGCCT |
| *Bax* | F: TGAAGACAGGGGCCTTTTTG  R: AATTCGCCGGAGACACTCG |
| *Bak* | F: CAACCCCGAGATGGACAACTT  R: CGTAGCGCCGGTTAATATCAT |
| *Bik* | F: ACGTGGACCTCATGGAGTG  R: TGTGTATAGCAATCCCAGGCA |
| *Bad* | F: AAGTCCGATCCCGGAATCC  R: GCTCACTCGGCTCAAACTCT |
| *Bim* | F: CCCGGAGATACGGATTGCAC  R: GCCTCGCGGTAATCATTTGC |
| *Bcl2* | F: GTCGCTACCGTCGTGACTTC  R: CAGACATGCACCTACCCAGC |
| *GAPDH* | F: CATGGCCTTCCGTGTTCCTA  R: TGCCTGCTTCACCACCTTCT |

**Table S4.** siRNA for EphA7 (Invitrogen)

| Name | sequence |
| --- | --- |
| si-EphA7-1 | CCCUAGGCUUGAUGUUGCCACACUU  AAGUGUGGCAACAUCAAGCCUAGGG |
| si-EphA7-2 | GCUGUAAAUGGAGUUUCGGACUUAA  UUAAGUCCGAAACUCCAUUUACAGC |
| si-EphA7-3 | UAGCAUUGGUUUCUGUCAAAGUGUA  UACACUUUGACAGAAACCAAUGCUA |

**Table S5**. guide RNA and detection primer sequences for *Zfp422* knockout in C2C12 (5’ to 3’)

| Name | sequence |
| --- | --- |
| sgZfp422_L | GCTTCGAGAAATACCCCCTT |
| sgZfp422_R | GCCCGCCAGCGACAGAAATG |
| Zfp422_detect_F | AGATTGAAGAGGTGCTAGTGCTG |
| Zfp422_detect_R | GGTGAGTTTTCACCCTGGCAT |

**Table S6**. Primer sequences for ChIP-enriched *EphA7* enhancer binding site by Zfp422-Myc (5’ to 3’)

| Name | sequence |
| --- | --- |
| EphA7-636K-F | CAGTAAATGGGAAATGTACAGTGTAGAAC |
| EphA7-636K-R | GGTGCGGGTTTCAAATTTTCTATATGTTTCAGT |

**Table S7**. guide RNA for generating *Zfp422^fl/+^* mice (5’ to 3’)

| Name | sequence | PAM |
| --- | --- | --- |
| 5’-end | GAATAGATAGGAATCTATGG AGG | AGG |
| 3’-end | TATCTGAGCTTGGACTATGT GGG | AGG |

**Table S8**. Primers for genotyping of *Zfp422^fl/+^* mice (5’ to 3’)

| No. | Name | Sequence | Product length (bp) |
| --- | --- | --- | --- |
| 1 | Zfp422-loxP-tF1 | CTGAATAAGACTCTTGGGAAACTTGAA | Flox： 325bp Wt： 234bp |
|  | Zfp422-loxP-tR1 | TATCTCTGCTAAACAGCATCTCAG |  |
| 2 | Zfp422-D-5in-tF1 | TCCCCAGGTCGGACAGTGAGAAAT | Flox：1066bp Wt： none |
|  | Common En2-R | CCAACTGACCTTGGGCAAGAACAT |  |
| 3 | ZMK2F4 | GCATCGCATTGTCTGAGTAGGTG | Flox：1166bp Wt： none |
|  | Zfp422-D-3in-tR1 | ACAGGATGCTCAGGGCATTCA |  |
| 4 | Zfp422-loxP-tF1 | CTGAATAAGACTCTTGGGAAACTTGAA | Flox：4511bp Wt： none |
|  | LAR3 | CACAACGGGTTCTTCTGTTA |  |

**Table S9.** Antibodies

| Name | Company | Catalogue |
| --- | --- | --- |
| eMyHC | DSHB | F1.652 |
| Myogenin | Millipore | MAB3876 |
| MyHC | Abcam | ab7784 |
| MyoD | Abcam | ab212662 |
| Pax7 | DSHB | PAX7 |
| Zfp422(IF) | biorbyt | orb313497 |
| Zfp422(WB) | Abcam | ab168151 |
| EphA7 | Abcam | ab133888 |
| MEF2C | CST | 5030 |
| Cleaved caspase-3 | CST | 9669 |
| Myc | CST | 2276 |
| Anti-mouse IgG | CST | 7076 |
| GAPDH | CST | 2118 |
| Anti-rabbit IgG (H+L), (Alexa Fluor® 488 Conjugate) | CST | 4412 |
| Anti-Mouse IgG (H+L), F(ab')2 Fragment (Alexa Fluor® 488 Conjugate) | CST | 4408 |
| Anti-rabbit IgG (H+L), F(ab')2 Fragment (Alexa Fluor® 555 Conjugate) | CST | 4413 |
| Anti-mouse IgG (H+L), F(ab')2 Fragment (Alexa Fluor® 555 Conjugate) | CST | 4409 |
